# What Does the Future Hold for Biomarkers of Response to Extracorporeal Photopheresis for Mycosis Fungoides and Sézary Syndrome?

**DOI:** 10.3390/cells12182321

**Published:** 2023-09-20

**Authors:** Oleg E. Akilov

**Affiliations:** Cutaneous Lymphoma Program, University of Pittsburgh, Pittsburgh, PA 15213, USA; akilovoe@upmc.edu

**Keywords:** biomarker, ECP, extracorporeal photopheresis, mycosis fungoides, Sézary syndrome

## Abstract

Extracorporeal photopheresis (ECP) is an FDA-approved immunotherapy for cutaneous T-cell lymphoma, which can provide a complete response in some patients. However, it is still being determined who will respond well, and predictive biomarkers are urgently needed to target patients for timely treatment and to monitor their response over time. The aim of this review is to analyze the current state of the diagnostic, prognostic, and disease state-monitoring biomarkers of ECP, and outline the future direction of the ECP biomarker discovery. Specifically, we focus on biomarkers of response to ECP in mycosis fungoides and Sézary syndrome. The review summarizes the current knowledge of ECP biomarkers, including their limitations and potential applications, and identifies key challenges in ECP biomarker discovery. In addition, we discuss emerging technologies that could revolutionize ECP biomarker discovery and accelerate the translation of biomarker research into clinical practice. This review will interest researchers and clinicians seeking to optimize ECP therapy for cutaneous T-cell lymphoma.

## 1. Introduction

Cutaneous T-cell lymphoma (CTCL) is a type of cancer that arises from the neoplastic amplification of cutaneous CD4+ memory T helper cells [1,2]. The two most common subtypes of CTCL are mycosis fungoides (MF) and Sézary syndrome (SzS), characterized by the gradual progression from patches to plaques, or to tumors and erythroderma, respectively [2,3]. Most patients with MF have an indolent clinical course, while SzS is a more aggressive and rare malignancy. Despite advances in CTCL, diagnosing and predicting the response to therapy remains a challenge. Thus, biomarkers are needed to identify patients, for better treatment targeting, and for monitoring the treatment response over time. 

Historically, the classification of the CTCL subtype has been based on the presence or absence of specific cellular markers on malignant T cells. For example, more than ten years ago, Campbell et al. [4] discovered that MF cells typically have markers of skin tissue-resident effector memory T cells (TRM). In contrast, SzS cells frequently have central memory T cells (TCM) phenotype [4]. Those differences were thought to account for the distinctions in the behavior and progression of MF and SzS [5]. However, recent studies have shown that the corresponding markers of malignant T cells may change during the course of the disease, not only between patients who carry the same diagnosis, but also with some level of heterogeneity within the same patient [6,7]. This heterogeneity poses a significant challenge for the accurate diagnosis and effective treatment of CTCL. As such, biomarkers are critically needed to help predict the disease progression and treatment response. 

The search for CTCL biomarkers is an active area of research. Extracorporeal photopheresis (ECP) has been the focus of biomarker discovery efforts since its approval by the FDA in 1988. While ECP can provide a complete response in some patients, identifying patients who would respond well to this therapy remains challenging. Diagnostic, prognostic, and disease state-monitoring biomarkers of ECP are urgently needed to better predict the response to therapy and guide treatment decisions. This review aims to analyze the current state of ECP biomarker research and outline future directions for biomarker discovery in CTCL. 

## 2. Biomarkers of MF and SzS

Biomarkers have become essential for diagnosing, predicting the response to treatment, and forecasting the disease prognosis [8,9,10,11,12,13]. However, diagnostic biomarkers for differentiating MF from SzS at the cellular level still need to be developed [12,14,15,16]. The absence of markers of neoplastic cells makes the histological diagnosis of early MF challenging and diagnosing SzS with just a skin biopsy is difficult [4]. Even the flow cytometry in SzS relies on the absence of maturation markers on neoplastic cells rather than on the detection of malignant markers, the search for which is still ongoing. In this section, we will discuss the current state of diagnostic and prognostic biomarkers for MF and SzS after characterizing the current state of knowledge of the carcinogenesis of MF and SzS (Table 1).

Studies have shown that while malignant cells in MF and SzS exhibit heterogenous phenotypes, there is significant transcriptomic overlap questioning the paradigm of the distinct expression of cell surface markers and chemokines on TCM in SzS and TRM cells in MF [4]. Indeed, both MF and SzS cells can present with phenotypic features of any of the major naïve or memory T cell subsets (based on the differential expression of CD62L [L-selectin] and CD45RA) but commonly overexpresses CCR4 and programmed cell death protein 1 (PD1), which suggests that malignant cells have a shared progenitor based on the functional state and delayed differentiation, rather than arising from a separate phenotype [4,17].

The circulation patterns of T cells are determined mainly via cell surface adhesion molecules and chemokine receptors [18]. Naïve Th cells circulate freely through the peripheral blood and secondary lymphoid tissues, partly due to the expression of the lymphoid-homing chemokine receptors L-selectin and CCR7, as well as the skin-homing chemokine receptors CCR10 and CCR4 [18]. Following activation, differentiated TCM cells continue to express L-selectin and CCR7 and retain the ability to circulate through the peripheral blood and secondary lymphoid tissues, whereas differentiated effector memory (TEM) cells lose the surface expression of L-selectin and CCR7 and are instead confined to peripheral tissues [18,19].

MF and SzS cell phenotypes exhibit significant heterogeneity [6,7], and although MF cells typically exhibit a TEM phenotype and are confined to the skin while SzS cells typically exhibit a TCM phenotype and move through the peripheral blood, skin, and lymph nodes, a recent study found no correlation between disease and the phenotype of the cell of origin [4,5,17]. Further, studies have shown characteristic ultraviolet (UV) light-associated mutations in both MF and SzS cells, suggesting that these cells acquire mutations in the skin before clonal proliferation [17]. At the same time, most likely this is a passenger mutation that has nothing to do with narrow band UVB (NB UVB), and clearly, previous treatment with phototherapy does not make the prognosis worse but quite the opposite [20]. Further mutations modulate the expression of surface molecules such as Fas (CD95) and Fas ligand (FasL), B-cell lymphoma 2 (Bcl-2), and Bax, contributing to immune system evasion and allowing the malignant cells to proliferate [11,12,15,21,22].

### 2.1. Diagnostic Biomarkers

Currently, the diagnoses of MF and SzS rely on the identification of specific (bio)markers that distinguish these diseases from other skin conditions. The identification of Sézary cells based on the detection of the monoclonal cells of the following phenotype: CD2+, CD3+, CD4+ CD5+, CD7−, CD8−, and CD26− in the peripheral blood [3,13,16]. The expansion of CD4 T cells, ≥90% of which have a loss of CD7, has a 93% accuracy to diagnose malignant cells [23]. However, differentiating MF from SzS at the cellular level remains challenging, although ten potential biomarkers such as CD25, PD-1, TOX, T-plastin, Twist1, and CD158k have been proposed to aid in differential diagnosis [8].

The diagnosis of MF is difficult since the early stages resemble other skin diseases where the accumulation of lymphocytes at the dermo-epidermal junction is also observed (e.g. psoriasis, various lichenoid processes, and eczema) [23]. The identification of the dominant transcription factors, such as T-bet and GATA3, in cells of interest has been suggested to differentiate Th1-mediated early-stage MF from Th2-mediated eczema and Th17-mediated psoriasis. However, the reproducibility of this approach has been challenging [24]. Alternatively, cytokines have been proposed as better markers to identify the microenvironment associated with each disease. For example, interleukin (IL)-12 has been found to be significantly increased in patients with early patch stage MF, as compared to psoriasis and eczema, and can be used diagnostically [25]. Alternatively, IL-4 and IL-10 are elevated in the skin of patients with SzS [26]. Elevated IL-13 has also been observed in the skin samples of patients with MF and SzS, in contrast to the skin samples of patients with psoriasis or normal skin [8].

It is increasingly recognized that a single diagnostic marker of malignant cells is unlikely to be identified, given the phenotypical plasticity and varying degree of the maturation of malignant cells. As such, the use of a multi-cytokine panel of RNA-based technologies may provide better diagnostic specificity and sensitivity than individual biomarkers. For example, Moerman-Herzog et al. (2020) identified a panel of genes (*ANK1*, *FCRL3*, *GATA6*, *HDAC9*, *IKZF2*, *PLS3*, *TIGIT*, *TOX*, *TWIST1*, and *STAT4*) that differentiated patients with SzS from the lymphocytic-variant of hypereosinophilic syndrome [27]. The transcriptome-based technologies may confirm the presence of malignant transformation but, even in this case, a multiple gene panel may be necessary to achieve optimal diagnostic accuracy.

In addition to using cell surface markers and cytokines to diagnose MF and differentiate from other dermatoses, the detection of a positive clone can also be helpful. T-cell receptor (TCR) gene rearrangement analysis can be used to detect a clonal population of T cells in the skin. By definition, MF is a malignancy of monoclonal T cells and an absence of a distinct clone points against the diagnosis of MF. The presence of a clonal population of T cells is not diagnostic of MF since it can be observed in other non-malignant dermatoses. Therefore, T cell clonality evaluation should be used in conjunction with other diagnostic methods to support or reject the diagnosis.

Additionally, the utilization of non-invasive techniques such as dermatoscopy for the detection and monitoring of cutaneous lymphomas complements this progress [28,29]. Integrating technologies like dermatoscopy into the diagnostic and monitoring processes can enhance the accuracy of disease assessment, aiding in early detection, treatment optimization, and the evaluation of therapeutic responses.

When considering the prognosis and treatment response, the tumor microenvironment (TME) might have an important impact on the behavior of malignant cells [30]. The TME includes the surrounding non-cancerous cells, blood vessels, extracellular matrix, and signaling molecules, and it is known to play a crucial role in the cancer progression and treatment response. In CTCL, the TME has been shown to be complex and dynamic, with various immune cells and cytokines present [30]. For example, regulatory T cells and myeloid-derived suppressor cells in the TME can inhibit antitumor immune responses [31]. In contrast, the increased levels of the stimulator of interferon genes (STING) have been associated with a better prognosis in MF patients [32]. Therefore, identifying biomarkers associated with the TME can aid in the diagnosis, prognosis, and treatment selection for CTCL patients.

### 2.2. Prognostic Biomarkers 

There has been increasing interest in the use of prognostic biomarkers that should predict disease behavior, accurately differentiating the indolent course from the aggressive disease [8,9,10,11,12,13]. An accurate prognosis of the disease course may influence the choice of therapy, allowing the optimization of the treatment pathway. The tumor microenvironment plays a significant role in cancer outcome. The cytokine milieu is important in shaping the immune response, with Th1-dominated environments being pro-inflammatory and perpetuating the anti-tumor response [33]. Th2-dominated environments, on the other hand, are anti-inflammatory and are associated with the promotion of IgE and eosinophils [34]. In the advanced stages of CTCL, there is a shift from a Th1 to a Th2 cytokine profile by tumor-infiltrating lymphocytes. Considerable attention has been given to the Th1/Th2 axis because the progression of CTCLs to an advanced stage is accompanied by a switch from a predominantly Th1 cytokine profile to a Th2 cytokine profile. Upon activation, Th cells become polarized, expressing specific cytokine profiles and contributing differentially to the immunological microenvironment. Th1-polarized cells are induced by IL-12 and have a pro-inflammatory effect mediated by interferon (IFN)-γ, while Th2-polarized cells are induced by IL-4 and stimulate antibody production primarily through the secretion of IL-4 [15,35].

Human skin is normally a predominantly Th1 environment [36], and an increase in Th2 cells is observed in advanced stage CTCL patients, including advanced tumors in MF [37]. This increase in Th2 cells results in an imbalance of Th1/Th2 and a lack of a pro-inflammatory Th1 immune response that impairs the immune system’s ability to launch an anti-tumor response against malignant cells [33]. This is exacerbated by high levels of IL-4, IL-7, and IL-13 present in the Th2 environment, which contribute to the overexpression of CD47 on SzS cells [38]. CD47 is a ubiquitously expressed transmembrane protein that inhibits phagocytosis and is involved in proliferation. The high expression of CD47 on SzS cells allows them to evade immune surveillance and phagocytosis, further exacerbating disease progression [26]. Additionally, the raised levels of IL-4, IL-7, and IL-13 cytokines correlate with poor overall survival in patients with SzS, suggesting that these cytokines may predict a poor response [26].

A recent paper sheds light on the question of how malignant CTCL T cells cause such profound inflammatory changes in the skin. Gluud et al. (2023) provided evidence that malignant T cells in CTCL secrete cytokines IL-13, IL-22, and oncostatin M (OSM) to induce JAK-STAT signaling in the surrounding keratinocytes; downregulate the filaggrin expression; and impair the skin barrier function [10]. This toxic effect on the skin is thought to be driven by the Th2-skewed phenotype of clonal T cells. The dysregulated cytokine milieu, in addition to the overexpression of CD47 on SzS cells, further contributes to the disease progression and poor response to the treatment.

Late-stage disease may also result in T cell exhaustion, which contributes to the Th1-to-Th2 shift. Exhausted Th1 cells lose their robust effector functions and have an altered transcriptional profile (TRM to TCM shift), leading to a loss of IL-2 production, followed by a loss of IFN-γ, TNF-α, and chemokine production, and the expression of multiple inhibitory receptors) [39]. In SzS, Th2-defining transcription factors including GATA3 and JunB are highly overexpressed, as well as integrin β1, proteoglycan 2, RhoB, and dual-specificity phosphatase 1, which serves as a positive feedback loop for self-perpetuating growth in the tumor microenvironment [40,41].

While certain clinical features, including a low malignant cell count and an intact CD8+ population which correlates with the indolent course [42], there is a growing interest in the use of multigene panels to enhance predictive accuracy. These panels allow for the assessment of the multiple genes and pathways involved in the disease progression of carcinogenesis and the TME and provide a more comprehensive understanding of disease behavior. So far, the use of multigene panels to predict the progression of MF and SzS has shown some promising results. For example, a study by Rindler et al. (2021) identified a 5-gene panel (*CXCR4*, *CD69*, *HSPA1A*, *ZFP36*, and *IL7R*) that was downregulated with MF progression [31]. 

In addition to multigene panels, cytokines may also serve as prognostic biomarkers for MF and SzS. Studies have shown that cytokines such as IFN-γ and IL-12, indicative of a Th1-dominated microenvironment, may be associated with an improved prognosis [37,43,44,45,46]. Conversely, cytokines such as IL-4, IL-7, and IL-13 have been linked to worse disease outcomes, at least partially due to their ability to stimulate the expression of cytoprotective CD47 (an independent marker of the disease progression) on the surface of malignant cells [26]. Additionally, IL-13 has been found to increase with the progressing MF and SzS disease stage [8]. Some studies demonstrated that the depletion of IL-2 has been associated with advanced disease, whilst other investigators have found no difference in the IL-2 level when compared to the healthy controls [26,37]. While the data on the prognostic value of other cytokines is less clear, continued research into their role in disease progression may provide further insight on the pathophysiology of MF and SzS.

## 3. Current Treatment Options

Current pharmacological treatments for CTCL include bexarotene, vorinostat, IFN-α and-γ, romidepsin, brentuximab vedotin, methotrexate, pralatrexate, gemcitabine, doxorubicin, alemtuzumab, mogamulizumab, and pembrolizumab. Psoralen and ultraviolet light (PUVA) therapy may also be used. Despite this therapeutic armamentarium, treatment options for CTCL remain challenging. None of those medications are curative, the response rates are moderate at best, the disease relapse is almost inevitable, and many medications carry serious side effects. Further, a visible response to treatment often takes several months to achieve, leading to significant stress on the patients. 

ECP remained a first-line immunotherapy for SzS approved by the FDA as a palliative treatment for CTCL in 1988 [47]. During ECP, approximately 5 × 10^9^ leukocytes are collected ex vivo via leukapheresis, treated with 8-methoxypsoralen (8-MOP), and photoactivated with UVA light, before being reinfused back into the patient [48,49]. ECP has been shown to induce malignant cell death [9,50] and activates monocytes and monocyte-Derived Cells (moDCs) [1,51,52] to produce a long-lasting immune response in a subset of patients. The safety and clinical benefits of ECP have been widely documented [1]. While the response rates to ECP are relatively high (50–70%), with complete resolution up to 17.6%, ECP can be resource-intense (device cost, single-use cassette, personnel training, and the necessity of infusion beds) and the time leading up to the response can be up to nine months [47,53]. 

One potential solution is better patient selection for targeted treatments. This approach could lead to improved clinical outcomes and reduced side effects. The identification of biomarkers that can predict the patient response to therapy is a critical area of research that could facilitate the proper treatment selection. Furthermore, novel technologies such as next-generation sequencing, gene expression profiling, and single cell RNA sequencing may identify the potential biomarkers associated with CTCL pathogenesis that were not clear in previous decades. 

## 4. ECP Molecular Mechanisms of Action and Biomarkers of Response 

**Figure 1:**
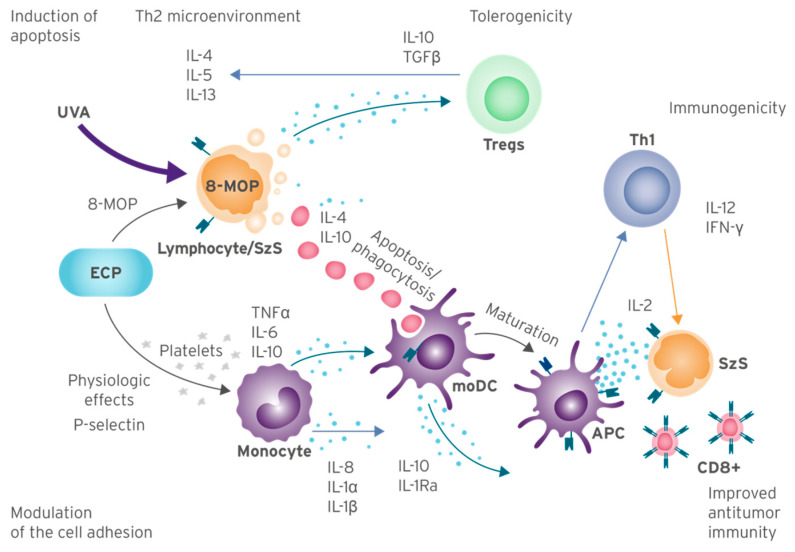
ECP Molecular Mechanisms of Action and Biomarkers of Response Overview. Summary of mechanisms of action from current knowledge of ECP, based on published studies. Abbreviations: MOP: 8-methoxypsoralen; APC: antigen-presenting cells; ECP: extracorporeal photopheresis; IL: interleukin; IL-1Ra: IL-1 receptor antagonist; INF-γ: interferon gamma; moDC: monocyte-Derived Cells; TNF-α: tumor necrosis factor alpha; Tregs: regulatory T cells; SzS: Sézary syndrome; UVA: ultraviolet A.

A full theory on how ECP produces a complete, lasting immune response to MF and SzS in some patients remains elusive; however, the understanding of its mechanisms of action has progressed significantly in recent years. Broadly speaking, these activities begin with the induction of apoptosis in malignant lymphocytes treated with 8-MOP and UVA light; simultaneously, monocytes are physiologically activated and presented to these apoptotic lymphocytes and, via phagocytosis, process the tumor-specific antigens which provide the basis for anti-tumor immunity. Additional effects, including the inhibition of inflammation via immune tolerance and the modulation of genes involved in cell adhesion and diapedesis, further work to restore healthy immune function [1,49,50,54]. Figure 1 provides an overview and the author’s proposal of the ECP mechanisms of action and potential biomarkers of response. 

### 4.1. Induction of Apoptosis 

A small percentage of lymphocytes collected ex vivo show signs of apoptosis almost immediately following the exposure to 8-MOP and UVA; the formation of monoadducts and covalent crosslinks of DNA causes morphological changes including the externalization of phosphatidylserine residues and the reversal of the Bax/Bcl-2 ratio [1,44,49,55,56,57,58,59]. Following this, a second late-stage apoptosis begins, characterized by the upregulation of the tumor suppressor gene *p53* and the modulation of Fas/FasL signaling which induces the activation-induced cell death (AICD) pathway [50]. By 72 h post-procedure, lymphocyte viability drops to around 12%, with the majority of directly treated lymphocytes becoming apoptotic and beginning to show signs of secondary necrosis [44]. Apoptosis appears to occur in almost all patients, regardless of the long-term immunogenic response [9].

It is not clear why malignant cell death leads to immunogenicity in some patients but not in others; however, there is a complex interplay of multiple factors involved. Fas/FasL signaling can induce both apoptosis and necrosis [60], whereas apoptosis is generally considered a silent event, necrotic cell death typically leads to inflammation [46]. Monocytes are specialized for chemotaxis and easily migrate to sites of inflammation [46]. Therefore, early necrosis may help to promote monocyte phagocytosis and thus provide both a source of antigens and an inflammatory environment which promotes the potent maturation of antigen-presenting moDCs. Necrotic and apoptotic cells, such as peripheral blood mononuclear cells (PBMCs), have demonstrated the release of cytokines such as IL-6, TNF-α, and MIP-1β [46].

### 4.2. Modulation of Cell Adhesion and Diapedesis

The transcriptional profiling of PBMCs one month post-ECP demonstrated the notable modulation of the genes responsible for cell adhesion and diapedesis [50]. The implications of this are unknown, as the current models of ECP therapy do not account for the changes in cell adhesion or diapedesis. However, several possibilities exist.

The interactions between moDCs and lymphocytes are mediated by both cytokines and cell adhesion molecules (Figure 1) [50]. A genomic analysis of post-ECP PBMCs suggests that ECP efficacy involves many biological pathways related to attachment, adhesion, diapedesis, and integrin signaling. This study demonstrated that these pathways were modified in the responders to ECP but not affected in non-responders [50]. The authors propose that the suppression of IL-1β-induced inflammation, involved in the adhesion and diapedesis pathways, may contribute to the mechanism of action of ECP [50].

ECP promotes the differentiation of antigen-presenting cells (APCs) via the direct engagement of monocytes by the ligands present on the activated platelets [61]. The proposed mechanistic sequence involves the binding of inactivated platelets to fibrinogen, followed by platelet activation and the expression of P-selectin. Monocytes then interact with the platelet-expressed ligands, leading to their efficient entry into the APC maturational pathway.

Transimmunization experiments suggest that incubating malignant lymphocytes with moDCs improve the probability of an immune response by enhancing the antigen loading into moDCs [62]. It is possible that the modulation of cell adhesion increases the likelihood of these interactions in vivo, where moDCs generally make up a very small percentage of leukocytes.

Integrins regulate T cell migration and have been implicated in tumor progression and metastasis, suggesting that ECP may reduce the tumor burden via the suppression of β1 and β2 integrins [50,63]. However, further research is needed to verify these initial results. While there are indicators of an integrin-mediated response to treatment in the literature [50], the importance of this mechanism is unknown. 

Significant progress has been made towards understanding the mechanisms of ECP and the pathogenesis of MF and SzS; however, vast areas of uncertainty remain. Validation of the model proposed in this article would contribute to an improved understanding of ECP and potentially improve the treatment pathway for patients with MF and SzS. The simultaneous measurement of multiple cytokines is necessary to capture the full complexity of these processes in individual patients [64].

### 4.3. Inhibition of Inflammation 

Apoptosis is generally a silent event producing no inflammation. The absence of an inflammatory environment results in the incomplete maturation of moDCs, resulting in a tolerogenic instead of an immunogenic impact. Immune tolerance is actioned primarily via the proliferation of Treg cells. A mechanism of immune tolerance has been linked to the stimulation of anti-inflammatory cytokines IL-10 and TGF-β by Treg cells [44,47,53].

As discussed, an overexpression of Th2 cytokines has been observed in CTCL patients [47,53]. The models suggest that successful treatment is characterized by a shift from a Th2-heavy microenvironment (IL-4, IL-10, and IL-13) to a Th1-heavy microenvironment (IL-12, IFN-γ, and TNF-α). However, the results in this area are sparse and have been conflicting [44]. For example, the transcriptional profiling of ECP-treated PBMCs noted that ECP efficacy might involve the suppression of the IL-1 signaling pathway [50]. It has been suggested that the effect ECP has on the Th1/Th2 pathways may depend on the initial Th1/Th2 imbalance of the disease state [47,53]. It is possible that ECP may invoke both beneficial pro- and anti-inflammatory effects in CTCL patients. There is undoubtedly a need for additional research in this area to better understand the impact of ECP on the tumor microenvironment.

### 4.4. Improved Anti-Tumor Immunity

Although almost all patients experience a modest reduction in malignant cell burden following treatment, relatively few patients go on to develop long-term immunity. The reasons for this are somewhat unclear, and future research might aim to characterize the specific conditions under which apoptotic lymphocytes are phagocytosed by moDCs in an immunogenic way.

In ECP, monocytes collected ex vivo undergo physiological changes that induce their development into moDCs [1,51], which play a crucial role in the phagocytosis of apoptotic lymphocytes, antigen presentation, and cytokine production. As the primary phagocyte found in the blood, monocytes and moDCs are responsible for most of the ECP-directed phagocytosis, and their mechanically active nature allows them to migrate to sites of inflammation to perform their immune functions. During phagocytosis, moDCs process antigens present on the dying cells, which are subsequently presented to the cytotoxic T cells bound to the moDC major histocompatibility complex (MHC), resulting in an anti-tumor immune response [46,51,58]. It is worth noting that the monocyte and DC markers used in the in vitro experiments cannot distinguish between these cell types, and it is known that moDCs possess a weak antigen presentation capacity. Therefore, it is possible that DC progenitors in the buffy coat could lead to a strong immunogenic response against the tumor cells. However, additional experiments are needed to prove this hypothesis.

Proinflammatory cytokines and phagocytosis markers including IL-6, TNF-α, and IFN-γ may indicate anti-tumor immunogenicity [46,51]. Importantly, anti-tumor immunity requires an intact cytotoxic CD8+ T cell population, as these cells are primed to induce apoptosis in malignant cells following activation via the association of the TCR with an APC MHC [65]. IL-2 acts as a growth and differentiation factor for activated cytotoxic T cells. Therefore, the measurement of this cytokine may indicate developing anti-tumor immunity; however, the specificity of such a measurement is unknown.

### 4.5. Biomarkers of Response 

Biomarkers of response should change during/after treatment, indicating the state of cancer entering remission or rendering resistance to the current therapy [8,9,10,11,12,13]. Several potential biomarkers have been investigated to predict the response in CTCL during ECP (Table 2), and studies have shown that circulating Tregs and IFN-γ+ cytotoxic T cells increase in the responders to ECP. Specifically, a study by Shiue et al. (2015) found that the responders to ECP significantly decrease Treg cells, which correlates with an increase in IFN-γ+ cytotoxic T cells [66]. These findings suggest that monitoring the levels of these cells during ECP treatment may be a valuable strategy to predict the response to therapy.

The cytokine response following ECP is controversial, with conflicting results reported in the literature. Some studies reported a shift towards a pro-inflammatory Th1 environment following ECP, with increased levels of cytokines such as IFN-γ and TNF-α [44,53]. In contrast, other studies have reported a shift towards an anti-inflammatory Th2 microenvironment with increased levels of cytokines such as IL-4 and IL-5 [11,47,53]. A study by McGirt et al. (2010) found that both TNF-α (pro-inflammatory) and IL-5 (anti-inflammatory) markedly increased six months after ECP in CTCL patients, suggesting that the cytokine response following ECP may depend on the initial Th1/Th2 balance [53]. However, further research is needed to validate these findings in larger patient populations and identify reliable biomarkers for predicting the response to ECP therapy.

## 5. Discussion

CTCL is a complex disease to diagnose and treat, with a lack of reliable biomarkers for the early diagnosis and effective monitoring of the treatment response. While research has primarily focused on diagnostic biomarkers [8,13,16,41,52], this remains challenging given the similarities between malignant and benign cells and offers little benefits clinically over a visual diagnosis. The simultaneous measurement of multiple cell surface markers has been used as a minimally accurate diagnostic test [13,50]; however, more specific and reliable biomarkers are needed. 

There is an unmet need for prognostic and biomarkers of response, which should vary significantly outside of the normal range under different disease conditions and capture the wide range of pathways affected by ECP. Current understanding suggests that a biomarker panel demonstrating anti-tumor immunogenicity in patients may provide the most accurate indication of the long-term response. This may include an increase in IFN-γ production in response to APC activity [43,48], a rise in IL-6 and TNF-α levels, and a decrease in IL-2 and IL-12 levels [30,31,32,33,37,43,44,51]. However, further research is needed to validate these markers and determine their concentrations associated with each stage of the disease.

Despite progress in understanding the mechanisms of action of ECP, the current models remain incomplete, and there is a large degree of uncertainty in the expected behavior of biomarkers of response. One promising avenue for research is the use of IL-1 and β integrins as indicators of the recently proposed therapeutic pathways in ECP [50,63,67], but further investigation is needed before their behavior can be confidently modeled. Additionally, the heterogeneity of CTCL and the lack of consensus on the diagnostic and response criteria present significant challenges that must be addressed.

In the realm of therapeutic management for cutaneous lymphomas, the significance of assessing patients’ quality of life (QoL) cannot be underestimated. Beyond the conventional clinical parameters, QoL monitoring serves as a pivotal marker in gauging treatment effectiveness and patient adherence [68]. Recognizing the potential impact of therapies on various aspects of daily life, including physical, emotional, and social well-being, QoL evaluation offers a comprehensive insight into the holistic effects of treatments. In the context of photopheresis and its inclusion in therapeutic regimens, tracking QoL provides a lens through which compliance to the treatment can be assessed [69]. A positive correlation between therapy compliance and improved QoL underscores the patient’s engagement with the treatment plan. As such, QoL monitoring not only enhances patient-centered care but also aids healthcare providers in tailoring interventions to optimize treatment outcomes.

The landscape of cutaneous lymphoma treatment has seen the emergence of combined therapeutic approaches that harness the strengths of different modalities to achieve improved outcomes. One such strategy involves the integration of cutaneous radiotherapy with other treatments, such as photopheresis. This combination leverages the precision of radiotherapy in targeting localized lesions while harnessing the systemic effects of treatments like photopheresis. The synergy between these modalities offers several advantages. Cutaneous radiotherapy excels in providing the rapid reduction of tumor burden and local symptom relief. Its ability to deliver targeted radiation promotes lesion regression and alleviates discomfort. On the other hand, photopheresis contributes to immune modulation, creating an environment conducive to long-term disease control. The immunomodulatory effects of photopheresis facilitate a systemic response against malignant cells, potentially preventing disease progression and recurrence. Moreover, the non-overlapping toxicities of these therapies reduce the risk of cumulative adverse effects, contributing to an improved therapeutic index. By combining the localized benefits of cutaneous radiotherapy with the systemic immune-enhancing effects of photopheresis, clinicians can optimize treatment approaches, achieving not only physical remission but also durable disease control and enhanced patient quality of life.

### 5.1. Limitations

A complete understanding of MF and SzS pathogenesis and the therapeutic mechanisms of ECP remains elusive. Suggestions for the role of other T cell subtypes, including Treg and Th17 cells, have been made, but supportive evidence remains weak. Furthermore, the immune system’s complexity means that considerable heterogeneity in the behavior of MF and SzS cells is to be expected, and there continues to be disagreement surrounding the classification of disease stages and progenitor cell types. All these factors combined reduce the certainty of the proposed models. Further studies are therefore crucial to build upon the current understanding and fill the unmet need for biomarkers of response in MF and SzS.

Although outside of the scope of this review, other biomarkers such as circulating micro RNAs and differentially expressed genes may offer greater accuracy; however, at present, they may be less practical as many clinics are not equipped with the appropriate measuring instruments.

### 5.2. Future Directions 

Artificial intelligence (AI) presents a significant opportunity to revolutionize biomarker discovery, particularly in the identification of novel and clinically relevant markers that might otherwise go unnoticed. AI algorithms can analyze large volumes of data from diverse sources such as genomics, proteomics, metabolomics, and imaging, and identify patterns and relationships that might not be evident to human investigators. Moreover, AI can aid in integrating the data from various sources and identifying biomarkers linked with specific disease subtypes or stages, thus making personalized medicine a reality. As AI technologies continue to advance, they are likely to become increasingly crucial in biomarker discovery and ultimately enhance patient outcomes. It would be beneficial to include single cell RNA sequencing as a future technology to track the cells originating from ECP treatments, settling in tissues, and comprehending the mechanism of action of ECP, as well as providing data for AI inputs. However, AI is not a panacea and requires appropriate validation and the careful consideration of ethical, legal, and social implications.

## 6. Conclusions

In conclusion, CTCL remains a challenging disease to diagnose and treat, requiring the identification of reliable biomarkers for the early diagnosis and effective monitoring of the treatment response. While significant progress has been made in identifying potential biomarkers, several challenges still need to be addressed, including the heterogeneity of the disease, the lack of consensus on the diagnostic and response criteria, and the need for advanced technologies and settings for biomarker identification. Future directions for biomarker discovery in CTCL should focus on developing standardized diagnostic and response criteria, using advanced technologies, and establishing large-scale biobanks and collaborative networks to identify robust and clinically applicable biomarkers.

## Figures and Tables

**Table 1:** Potential Biomarkers of MF and SzS.

Type	Diagnostic Biomarkers	Prognostic Biomarkers
Cytokines (individual and groups)	IL-3	Shift from a Th2-heavy microenvironment (IL-4, IL-10, IL-13) to a Th1-heavy microenvironment (IL-12, IFN-γ, TNF-α)
	IL-4	IL-4
	IL-10	IL-7
	IL-12	IL-13
		IL-22
		IFN-γ
		IL-12
		IL-2
		Oncostatin M
Cell markers, transcription factors, and other proteins	CD25	CD47
	CD158K	
	PD-1	
	GATA-3	
	T-bet	
	TOX	
	Twist1	
	T-plastin	
	STING in the TME	
Genes panels	Moerman-Herzog et al. (2020) gene panel (*ANK1*, *FCRL3*, *GATA6*, *HDAC9*, *IKZF2*, *PLS3*, *TIGIT*, *TOX*, *TWIST1*, *STAT4*)	Rindler et al. (2021) 5-gene panel (*CXCR4*, *CD69*, *HSPA1A*, *ZFP36*, and *IL7R*
Cell populations	CD2+, CD3+, CD4+ CD5+, CD7−, CD8−, CD26− phenotype in the peripheral blood	T cell exhaustion (loss of IL-2, IFN-γ, TNF-α, and chemokine production)
	CD4 T cells (CD7 loss)	Low malignant cell count
	Positive clone (TCR gene rearrangement analysis)	Intact CD8+ population
	Treg and myeloid-derived suppressor cell levels in the TME	

Abbreviations: IFN-γ: interferon gamma; IL: interleukin; MF: mycosis fungoides; STING: stimulator of interferon genes; SzS: Sézary syndrome; TCR: T-cell receptor; TME: tumor microenvironment; TNF-α: tumor necrosis factor alpha; Treg: regulatory T cells.

**Table 2:** Potential Biomarkers of Response to ECP treatment.

Type	Potential Biomarkers of Response
Cytokines (individual and groups)	Shift from a Th2-heavy microenvironment (IL-4, IL-10, IL-13) to a Th1-heavy microenvironment (IL-12, IFN-γ, TNF-α)
	Proinflammatory cytokines and phagocytosis markers including IL-6, TNF-α, and IFN-γ
	IL-1
	IL-2
	IL-5
	IL-4
	TNF-α
Cell markers, transcription factors, and other proteins	β1 and β2 integrins
Cell populations	Intact cytotoxic CD8+ T cell population
	Circulating Tregs and IFN-γ+ cytotoxic T cells

Abbreviations: IFN-γ: interferon gamma; IL: interleukin; TNF-α: tumor necrosis factor alpha; Treg: regulatory T cells.

## Data Availability

No new data were created or analyzed in this study. Data sharing is not applicable to this article.
